# Maturation of Sensori-Motor Functional Responses in the Preterm Brain

**DOI:** 10.1093/cercor/bhv203

**Published:** 2015-10-21

**Authors:** Alessandro G. Allievi, Tomoki Arichi, Nora Tusor, Jessica Kimpton, Sophie Arulkumaran, Serena J. Counsell, A. David Edwards, Etienne Burdet

**Affiliations:** 1 Department of Bioengineering; 2 Division of Brain Sciences, Department of Medicine, Imperial College of Science, Technology and Medicine, London SW7 2AZ, UK; 3 Centre for the Developing Brain, Division of Imaging Sciences and Biomedical Engineering, King's College London, King's Health Partners, St Thomas’ Hospital, LondonSE1 7EH, UK

**Keywords:** development, neonate, sensori-motor, task fMRI

## Abstract

Preterm birth engenders an increased risk of conditions like cerebral palsy and therefore this time may be crucial for the brain's developing sensori-motor system. However, little is known about how cortical sensori-motor function matures at this time, whether development is influenced by experience, and about its role in spontaneous motor behavior. We aimed to systematically characterize spatial and temporal maturation of sensori-motor functional brain activity across this period using functional MRI and a custom-made robotic stimulation device. We studied 57 infants aged from 30 + 2 to 43 + 2 weeks postmenstrual age. Following both induced and spontaneous right wrist movements, we saw consistent positive blood oxygen level–dependent functional responses in the contralateral (left) primary somatosensory and motor cortices. In addition, we saw a maturational trend toward faster, higher amplitude, and more spatially dispersed functional responses; and increasing integration of the ipsilateral hemisphere and sensori-motor associative areas. We also found that interhemispheric functional connectivity was significantly related to ex-utero exposure, suggesting the influence of experience-dependent mechanisms. At term equivalent age, we saw a decrease in both response amplitude and interhemispheric functional connectivity, and an increase in spatial specificity, culminating in the establishment of a sensori-motor functional response similar to that seen in adults.

## Introduction

The human brain undergoes a dramatic and rapid sequence of maturation in the third trimester of gestation as the cortex rapidly increases in size through folding, and afferent axons sprout through the transient subplate zone (de Graaf-Peters and Hadders-Algra 2006; Kostovic and Jovanov-Milosevic 2006; Kostovic and Judas 2010). The critical importance of this period for brain development is emphasized by the effects of preterm birth (delivery at <37 weeks gestation) which engenders a specific and persistent pathological phenotype of impaired regional growth and white matter integrity, reduced cortical folding, and impaired thalamo-cortical structural connectivity (Boardman et al. 2010; Rathbone et al. 2011; Ball et al. 2013).

Relative to other neural systems which also allow environmental interaction, the sensori-motor system begins to mature early in gestation and is highly functional throughout human in-utero life (de Vries et al. 1982). Although reflex movements can be demonstrated in fetuses as young as 7–8 weeks gestation, intracerebral structural involvement is not seen until 18–20 weeks gestation, when cortical plate lamination and sulcation form the anatomical substrate of the primary motor cortex (Marin-Padilla 1970; Molliver et al. 1973; Tawia 1992). Immature somatosensory evoked responses, cortical hemodynamic responses to painful stimulation, and electromyography responses to transcranial magnetic stimulation can all be reliably identified in preterm infants from the equivalent period to the early third trimester of gestation (Hrbek et al. 1973; Taylor et al. 1996; Eyre et al. 2000; Bartocci et al. 2006; Slater et al. 2006). The altered patterns of sensory stimulation experienced by preterm infants during their ex-utero life may therefore have important implications including contributing to the aforementioned structural brain abnormalities; particularly when considered in the context of animals models which suggest that altered tactile stimulation during early life may permanently alter somatosensory development through activity-dependent mechanisms (Fox 1992).

However, little is known about functional brain development during this critical time and, moreover, how it may be influenced by external factors. Blood oxygen level–dependent (BOLD) functional magnetic resonance imaging (fMRI) can provide visualization of functional brain activity during tasks and at rest, with excellent whole-brain spatial specificity (Ogawa et al. 1992). These techniques have revealed that as resting-state networks (RSNs) mature during the preterm period, long-range functional connectivity systematically increases, eventually achieving an adult-like spatial representation at term equivalent age (Doria et al. 2010). Of importance however, the neural origin of resting-state activity is still not fully understood; and the accepted topography of mature functional anatomy cannot be assumed throughout early brain development. Thus, task-based fMRI studies in infants are essential to provide precise characterization of both the spatial localization of activity and the dynamic relationships within specific functional systems; and may be more representative of functional integrity than resting-state fMRI alone. Although task-based fMRI has been used to demonstrate functional responses in the neonatal brain in the visual (Born et al. 1996), auditory (Anderson et al. 2001), olfactory (Arichi et al. 2013), and somatosensory (Erberich et al. 2003, 2006; Heep et al. 2009; Arichi et al. 2010) systems (reviewed in Seghier and Huppi 2010), it has not been used to systematically characterize the maturation of responses during the preterm period. We have previously developed and validated a set of fMRI-compatible tools including a computer-controlled robotic hand and wrist stimulator (Arichi et al. 2010; Allievi et al. 2013) and a preterm-specific hemodynamic response function (HRF) (Arichi et al. 2012), which have allowed us to identify robust patterns of positive BOLD functional activity in preterm infants.

Here we aimed to use this methodology to build further on our previous work by characterizing the temporal and spatial evolution of sensori-motor functional responses in 57 infants aged between 30 and 43 weeks postmenstrual age (PMA) (equivalent to the mid-third trimester of gestation to full term); and further explore the possible effects of premature exposure to the ex-utero environment on this process. For the first time in human infants, we also aimed to characterize the intracerebral correlates of spontaneous motor behavior using fMRI. We hypothesized that increasing PMA would be associated with faster and more spatially complex functional responses and that maturation would be significantly altered by experience.

## Materials and Methods

The work was approved by the National Health Service ethics committee, and written parental consent was obtained for all subjects prior to data collection.

### Study Population

Subjects were recruited from the Neonatal Intensive Care Units (NICU) at the Queen Charlotte and Chelsea Hospital London and St Thomas' Hospital London over a 3-year period between 2011 and 2013. All of the infants were recruited as part of a larger longitudinal study of the effects of prematurity on early brain development using MRI scanning, and were scanned only for research reasons with no clinical indication for imaging. The population for this part of the study consisted of a total of 57 infants (range 30 + 2 to 43 + 2 weeks PMA), of which the majority (55/57) had been delivered prematurely. Five of the infants in the study population had data collected at 2 time-points (during the preterm period and at term equivalent age), resulting in a total of 62 datasets. Prior to enrollment, all infants were reviewed by an experienced pediatrician to assess their clinical status, and identify any clear contraindications for MRI scanning. To reduce heterogeneity within the study group, infants were excluded if they required any respiratory support during scanning, and if they were known to have a history of brain pathology such as extensive intraventricular hemorrhage (grade 3 with ventricular dilatation; grade 4 with parenchymal extension), birth asphyxia, focal intracerebral lesions affecting the parenchyma or white matter (such as infarction, overt hemorrhage, or multiple punctate white matter lesions), severe hydrocephalus, or congenital brain malformations.

### Image Acquisition

Images were acquired using a 3-Tesla Philips Achieva MRI systems (Best, the Netherlands) and an 8-channel phased array head coil, located on the NICUs at the Queen Charlotte and Chelsea Hospital, London; and St Thomas' Hospital, London. Prior to imaging, a neonatal neurological examination was performed by an experienced practitioner (Mercuri et al. 2005); and clinical (antenatal, birth, and postnatal care) and demographic details were recorded from the clinical notes. Oral sedation (chloral hydrate 30–50 mg/kg dose) was administered approximately 20 min before scanning to only 2 of the 42 infants less than 37 weeks PMA, but to the majority (15/20) of infants at term equivalent age in order to decrease head motion and thus improve image quality, and reduce possible infant distress in the unfamiliar scanner environment. Sedation was only offered to families following clinical review to ensure that there were no contraindications for its use (such as severe gastroesphageal reflux or respiratory distress), and given following the parents' written consent. Infants who did not receive sedation were fed immediately prior to the scan, swaddled to encourage natural sleep, and immobilized using a vacuum-evacuated bag (Med-Vac, CFI Medical Solutions, Fenton, MI, USA). Hearing protection was applied in the form of molded dental putty in the external auditory meatus (President Putty, Coltene Whaledent, Mahwah, NJ, USA) and adhesive earmuffs (MiniMuffs, Natus Medical, Inc., San Carlos, CA, USA). All sessions of image acquisition were attended by a clinician trained in neonatal resuscitation, and physiological parameters (oxygen saturations, heart rate, and axillary temperature) were monitored throughout the imaging session. There were no adverse incidents during the data acquisition period related either to the sedation or MR imaging process. High-resolution structural 3D magnetization-prepared rapid gradient echo (MPRAGE) *T*_1_-weighted, turbo spin-echo (TSE) *T*_2_-weighted, and diffusion-weighted images were acquired for all infants for clinical review (sequence parameters can be found in Merchant et al. (2009)). In addition, the white matter appearance on structural images was scored by a neonatal neuroradiologist for each (prematurely born) infant at term equivalent age using a widely used qualitative system (Inder et al. 2003). fMRI data were collected from all infant subjects with a gradient echo echo-planar imaging (EPI) sequence lasting 6 min and 34 s with parameters: repetition time (TR) 1500 ms; echo time 45 ms; flip angle 90°; resolution (*x* × *y* × *z*) 2.5 × 2.5 × 3.25 mm (slice gap: 0.75 mm); total 256 volumes; SENSE factor 2.

### Passive Motor Task fMRI Experimental Design

A safe and reproducible pattern of proprioceptive stimulation was delivered to the infant subjects using a fully automated, custom-made fMRI-compatible wrist robot (Allievi et al. 2013). Wrist flexion/extension was chosen as a specific joint movement which could be readily induced without causing discomfort to the infant, and would minimize other tactile effects such as skin stretching, which could confound the elicited cortical responses. Prior to each scanning session, the robotic interface was secured to the right forearm and hand of each subject. During fMRI data acquisition, pneumatic actuation of the small device-mounted piston was used to guide gentle wrist movements, which were monitored in real time using a fiber-optic position sensor (see Supplementary Movie M1) (Allievi et al. 2013). The timing and pattern of proprioceptive stimulation was recorded and controlled during each experiment through full integration of the robotic interface to a system control unit in the scanner control room; composed of pneumatic equipment controlled via a National Instruments data acquisition card interfaced to custom software on a standard PC (Labview National Instruments, Austin, TX, USA). The synchronization between stimulation and fMRI image acquisition was ensured via detection of the transistor-to-transistor logic pulse emitted by the scanner with each TR. A simple block paradigm of periodic proprioceptive stimulation was used consisting of alternating 24-s blocks of rest and 24 s of 0.33-Hz sinusoidal passive wrist flexion/extension. The stimulation paradigm and parameters were selected based on those used previously to successfully induce statistically significant clusters of BOLD signal response in both preterm and term infants (Arichi et al. 2010; Allievi et al. 2013).

### fMRI of Spontaneous Motor Behavior

For studying spontaneous motor behavior (“active” motor condition), the actuating piston was disengaged, thus significantly reducing the drag and inertia related to rotation of the device's arms and therefore allowing the infant to easily make spontaneous movements of the right wrist with minimal resistance (Allievi et al. 2013). fMRI data were then acquired with identical acquisition parameters as the passive motor task, but with no pattern of induced motor stimulation. For every spontaneous wrist movement identified during the acquisition period, the displacement was precisely measured via the fiber-optic goniometer incorporated into the robotic device, sampled at a frequency of 100 Hz, and recorded using the custom Labview software program. The measured displacement was then converted into absolute wrist velocity and averaged for each TR of image acquisition, thus creating a precise time-locked measure suitable for later fMRI data analysis.

### fMRI Data Analysis

Data analysis was performed using FSL (FMRIB's Software library, Oxford, UK, www.fmrib.ox.ac.uk/fsl) (Smith et al. 2004). Functional datasets were visually scrutinized for evidence of sustained head motion, severe image distortion, and field-of-view misalignments, and corrupted data were discarded accordingly. The remaining datasets were truncated such that epochs affected by isolated bursts of head motion exceeding a total absolute displacement of 1.25 mm (equivalent to half-a-voxel in the acquisition plane) as calculated by MCFLIRT (FSL's intramodal motion correction tool) were deleted. Following deletion, passive motor task datasets were only included in the final analysis if containing a minimum of 128 consecutive volumes, ensuring that a minimum of 4 contiguous blocks of stimulation and rest would always be maintained. In the spontaneous motor behavior condition, epochs of data were discarded if the detected spontaneous wrist movements occurred simultaneously with head movement; and the entire dataset was discarded if there were less than 3 spontaneous movements during the acquisition period. This systematic removal of motion-corrupted data was implemented to minimize the possible detrimental impact of false correlations in signal caused by motion artifact on fMRI analysis despite standard registration and motion estimate regression techniques (Power et al. 2012; Satterthwaite et al. 2012; Van Dijk et al. 2012). This issue is of additional significance in neonatal fMRI data, which is inherently more susceptible to the “spin-history” signal effects caused by head motion, due to the relatively long *T*_1_ of neonatal brain tissue (Friston et al. 1996; Williams et al. 2005).

Each of the remaining patient datasets were then preprocessed using the standard pipeline as implemented in FEAT (fMRI Expert Analysis Tool, v5.98), which entails rigid-body head motion correction (using MCFLIRT), slice-timing correction, nonbrain tissue removal, spatial smoothing (Gaussian filter of full-width half-maximum [FWHM] 5 mm), global intensity normalization, and high-pass temporal filtering (cutoff 50 s) (for full details see Smith et al. (2004); Arichi et al. (2010)). Signal artifact related to residual motion and physiological noise (such as cardiovascular pulsation and respiratory motion) were also removed by performing additional data de-noising with MELODIC (Model-free FMRI analysis using Probabilistic Independent Component Analysis [PICA, v3.0]) (Beckmann and Smith 2004).

Time-series statistical analysis in FEAT was carried out using FMRIB's improved linear model with local autocorrelation correction (Woolrich et al. 2001). A general linear model (GLM) was used to perform a univariate (voxel-wise) fitting of the observed data to a linear combination of our explanatory variables: the boxcar functions representing the time course of proprioceptive stimulation (or the timing of wrist movements in the spontaneous motor behavior condition) and the detected head motion parameters as confound regressors (6 degrees of freedom [DOF]; translations and rotations). An optimized set of basis functions derived from an age-appropriate HRF for both preterm and term equivalent infants (and its temporal and dispersion partial derivatives) were convolved with the aforementioned explanatory variables in the GLM in order to capture the expected range of latency and duration of the evoked functional responses (Arichi et al. 2012). The calculated *t*-statistical image was then converted to a *z*-statistical image (and combined in a *F*-test to identify the full model fit derived from each of the basis functions), and a threshold of 2.3 with a corrected cluster significance level of *P* < 0.05 was then used to generate spatial maps of activated voxels on an individual subject level. To allow better anatomical localization of the identified functional activity, all of the filtered raw data and their corresponding activation maps were then registered to the individual subject's high-resolution structural *T*_2_-weighted image using a 6 DOF rigid-body registration as implemented in FMRIB's linear image registration tool (FLIRT v5.5) (Jenkinson and Smith 2001).

The temporal characteristics and percentage BOLD signal change of the functional activity related to the passive task condition were then studied within a region of interest (ROI), defined as voxels within the top quartile of *z*-score within the identified cluster of activity in the contralateral primary somatosensory cortex. The lag time to reach the response peak amplitude was identified by calculating the mean of the parameter estimate values derived from the GLM analysis for each of the basis functions within the ROI. These values were then multiplied by each of the original basis functions, summed to identify a subject-specific HRF, and the lag time identified as the time the maximum of the positive peak was reached. Percentage signal change within the ROI was calculated by multiplying the parameter estimate by the peak-to-peak height of the EV model, and dividing by the mean signal across the whole acquisition period (Mumford 2007).

### Functional Connectivity Analysis

Functional connectivity was assessed by calculating the correlation between the left and right peri-rolandic regions (encompassing both the primary motor cortex anterior to the central sulcus and the primary somatosensory cortex posterior to the central sulcus) during the period of functional stimulation in the passive motor task. To avoid data “double-dipping,” the peri-rolandic regions for this analysis were defined by anatomical masks (delineated in standard template space (Doria et al. 2010)) registered to the individual subject's native space (Kriegeskorte et al. 2009). The Pearson's partial correlation coefficient between the mean BOLD signal time-series within the left and right peri-rolandic regions was then calculated controlling for the whole-brain mean signal and the 6 detected head motion parameters, using tools implemented in the statistics toolbox of Matlab (The Mathworks, Natick, MA, USA). The calculated partial correlation coefficients were then normalized by conversion to a *z*-statistic using the Fisher's transformation. To assess the relationship between developmental changes in interhemispheric functional connectivity and ex utero exposure, correlation of each subject's connectivity measure was then assessed with their PMA and their postnatal age (PNA) in days.

### Higher Level Group Analysis

Individual subject *F*-test maps were co-aligned to an age-specific spatio-temporal neonatal atlas using FSL's nonlinear image registration tool (FNIRT v2.0) (Andersson et al. 2010; Serag et al. 2012). To allow use of the full lower level *F*-test maps and due to the data's unknown noise distribution, higher level group analysis was then performed using permutation testing as implemented in FSL Randomise (v2.1) (Nichols and Holmes 2002). Preterm subjects were grouped by their PMA into three groups (31–32 + 6 weeks; 33–34 + 6 weeks; 35–36 + 6 weeks), with a fourth group consisting of infants at term equivalent PMA (37–44 weeks). Nonparametric *t*-tests (correcting for the gestational age at birth) with threshold-free cluster enhancement were then used to identify significant clusters with a family-wise error correction to correct for multiple comparisons and a *P*-value threshold of *P* < 0.05 (Hayasaka and Nichols 2003; Nichols and Hayasaka 2003; Smith and Nichols 2009). Using this approach, the possible confounding effects of sedative medication on the identified responses were also explored in a subset of 10 patients (5 of whom had received sedation for data collection) using an unpaired *t*-test (subjects were matched between groups by PMA at scan). The possible effects of ex utero exposure during the preterm period were further studied by assessing its effect on the spatial pattern of the identified functional responses, using the PNA in days as a linear variable (correcting for PMA at scan).

## Results

Functional MRI data was successfully collected from 47 of the 62 infants studied. Data were discarded in 11 infants due to excessive head motion throughout the period of image acquisition which could not be resolved with standard correction measures; in 3 infants due to the identification of unexpected pathology (one infant with subependymal heterotopia, one with severe punctate white matter lesions, and one with severe cerebellar hemorrhage); and in 1 infant due to severe image artifact associated with the EPI acquisition sequence. The final study group therefore consisted of 9 infants studied between 31 and 32 + 6 weeks PMA at the time of MRI scan (median age: 32 + 1 weeks PMA); 13 infants between 33 and 34 + 6 weeks PMA (median age: 34 + 1 weeks PMA); 10 infants between 35 and 36 + 6 weeks PMA (median age: 35 + 6 weeks PMA); and 15 infants at term equivalent age (between 37 and 44 weeks PMA) at the time of data acquisition (median age: 41 + 1 weeks PMA) (full study population details are presented in Table 1). The majority of preterm-born infants at term equivalent age (13/15) had only a mildly abnormal white matter appearance on structural imaging, and one infant had a moderately abnormal appearance (the other infant was delivered at term and therefore not suitable for scoring). All of the infants included in the final study population had a normal and age-appropriate clinical neurological examination.

**Table 1:** Demographic details of the study population

Patient group	Number of subjects	Sex (male/female)	Postmenstrual age at scan in weeks + days (median, ranges)	Gestational age at birth in weeks + days (median, ranges)	Birth weight in grams (median, ranges)	Birth occipito-parietal head circumference in centimeters (median, ranges)
31–32 weeks	9	(4/5)	32 + 1 (31 + 0 to 32 + 4)	29 + 5 (27 + 3 to 30 + 5)	1210 (695 to 1650)	27 (24.6 to 29.9)
33–34 weeks	13	(4/9)	34 + 2 (33 + 3 to 34 + 5)	31 + 3 (27 + 1 to 33 + 2)	1450 (1025 to 2100)	28.1 (26 to 30.5)
35–36 weeks	10	(4/6)	35 + 6 (35 + 0 to 36 + 1)	34 (29 + 5 to 35 + 6)	1640 (850 to 2160)	29.0 (26 to 31.5)
Term equivalent (37–44 weeks)	15	(5/10)	41 + 1 (37 + 6 to 43 + 2)	29 + 6 (25 + 3 to 39 + 5)	1350 (545 to 3520)	28.25 (22.7 to 33.5)

### Passive Motor Task fMRI Results

**Figure 1.**
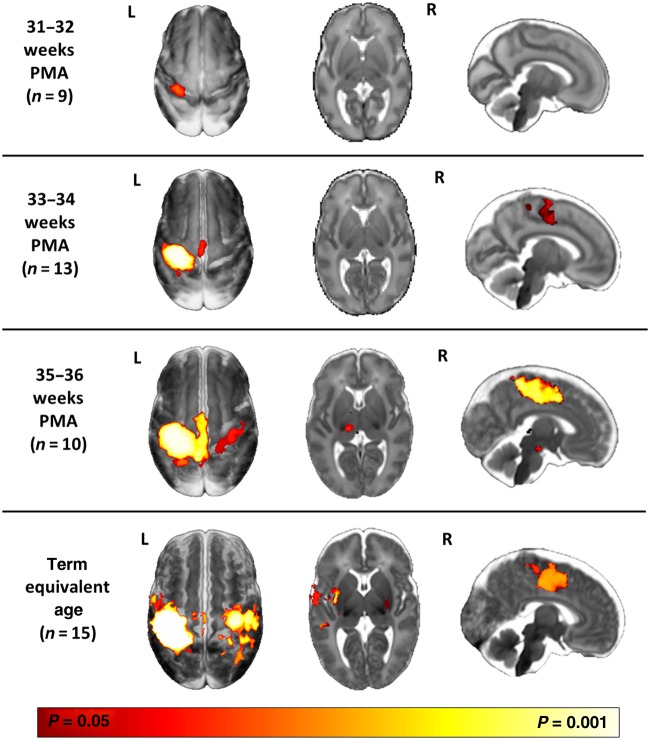
Evolution of sensori-motor functional responses induced by passive movement of the right wrist during. Following right wrist movement, localized functional activity was identified in all infants in the contralateral (left) primary somatosensory cortex. Functional responses can be seen to progress from a contralateral only pattern in the youngest infants at 31–32 weeks postmenstrual age (PMA) (top row, *n* = 9); to include the midline supplementary motor area (SMA) at 33–34 weeks (second row, *n* = 13); and the ipsilateral peri-rolandic cortex and thalamus at 35–36 weeks (third row, *n* = 10). At term equivalent age (37–44 weeks; fourth row, *n* = 15), a mature adult-like activation pattern is seen in the bilateral peri-rolandic regions, basal ganglia, SMA, and contralateral opercular cortex/secondary somatosensory cortex. The images show the results of one-sample *t*-test performed using permutation testing and corrected for family-wise error (FWE) overlaid on an age-specific 4D brain *T*_2_-weighted brain atlas (Serag et al. 2012).

Following induced flexion and extension movements of the right wrist, well-localized clusters of positive BOLD contrast activation were identified in the primary somatosensory cortices of all of the infants studied across the full range of ages (see Fig. 1). In the youngest study group (31–32 weeks PMA), an exclusively contralateral (left) and unilateral pattern of activation was seen over the region of the peri-rolandic cortex (encompassing both the primary motor cortex and the primary somatosensory cortex). Group analysis at 33–34 weeks PMA, similarly identified an exclusively contralateral, but more spatially dispersed pattern of functional activity in the peri-rolandic cortex with additional extension into the midline supplementary motor area (SMA). At 35–36 weeks PMA, the identified functional responses were further dispersed across the contralateral peri-rolandic cortex, with widespread involvement of the SMA. In addition, activity was also seen across the entire width of the ipsilateral (right) peri-rolandic cortex and in the contralateral thalamus. At the conclusion of the third trimester of gestation (37–44 weeks PMA or term equivalent age), a clear pattern of bilateral functional responses was seen, with clusters of activity visible in both the left and right peri-rolandic cortices. Identified clusters in the SMA and ipsilateral peri-rolandic cortex were more spatially localized than in the preceding weeks, with additional activation also seen bilaterally in the basal ganglia and left opercular cortex (secondary somatosensory cortex). A *t*-test analysis did not identify any significant difference in the spatial pattern or amplitude of functional responses between a group of infants who received sedative medication prior to scanning (median PMA: 40 + 4 weeks, range 35 + 4 to 41 + 1 weeks PMA, mean BOLD signal change 0.67%) and a group of infants who did not (median PMA: 38 + 4 weeks, range 35 + 4 to 41 + 0 weeks PMA, mean BOLD signal change 0.60%).

**Figure 2.**
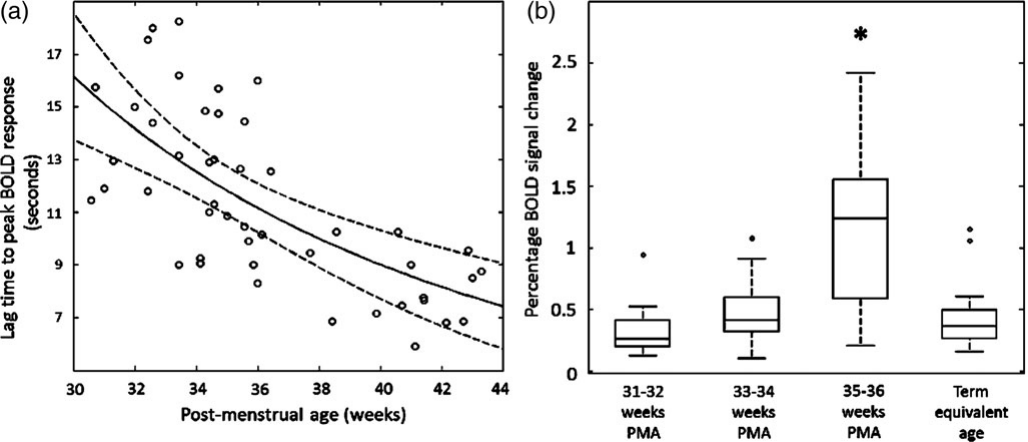
Maturational changes in the temporal characteristics and amplitude of the identified functional brain activity (*a*). The lag time to the positive peak of the identified response decreases systematically with increasing PMA (data shown with robust regression line with prediction error bands). (*b*) The amplitude of BOLD signal change within the identified clusters of activity are significantly higher at the conclusion of the third trimester (35–36 weeks PMA) in comparison to younger infants and those at term equivalent age (line represents data median; box represents data lower and upper quartile; *P < 0.05 Mann–Whitney *U*-test with Holm–Bonferroni correction).

In keeping with findings described in both small animals and our previous work with human preterm infants (Colonnese et al. 2008; Arichi et al. 2012), a clear maturational trend was seen in the temporal characteristics of the functional responses, with increasing PMA associated with a shorter lag time to achieve the positive peak amplitude of the de-convolved HRF within the identified clusters of activity in the contralateral hemisphere (Fig. 2*a*). The amplitude of the identified functional responses was seen to increase during the preterm period, with the maximum and significantly highest BOLD percentage signal change detected in the late preterm period (35–36 weeks PMA) (*P* < 0.01 Mann–Whitney *U*-test with Holm–Bonferroni correction for multiple comparisons) (Fig. 2*b*). The response amplitude then decreased at term equivalent age, such that it was not significantly different from that seen earlier in the third trimester.

### fMRI of Spontaneous Motor Behavior

**Figure 3.**
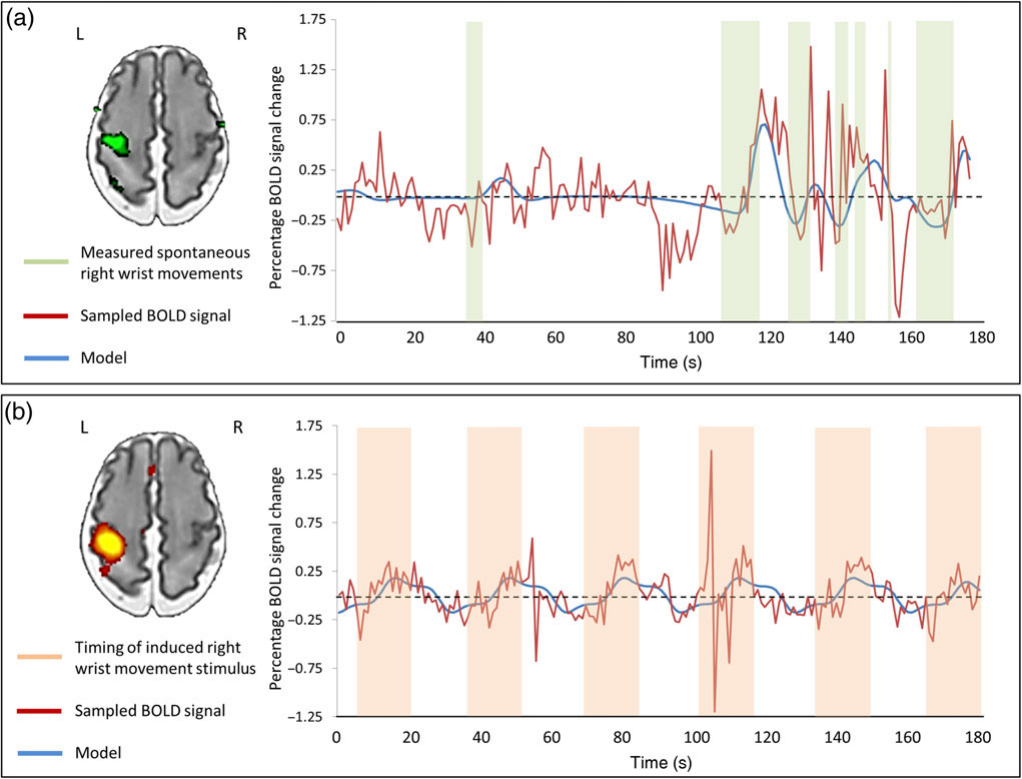
Spontaneous limb movements in the preterm brain are associated with functional brain activity in the peri-rolandic cortex. (*a*) Using a precise fiber-optic position sensor, spontaneous “active” right wrist movements during image acquisition were recorded (green blocks) and convolved with an age-specific hemodynamic response function (HRF) to model (blue trace) the acquired BOLD signal (red trace). In this example, preterm infant (32 + 3 weeks PMA), spontaneous movements of the right wrist were significantly correlated with the mean BOLD signal in the left peri-rolandic cortex (green cluster). (*b*) This same approach can also be used to model brain responses induced by “passive” right wrist movement (orange blocks), with the measured BOLD signal closely fitting the design model. In the same infant, this allows the identification of a localized cluster of functional activity (red–yellow) in the left peri-rolandic region.

**Figure 4.**
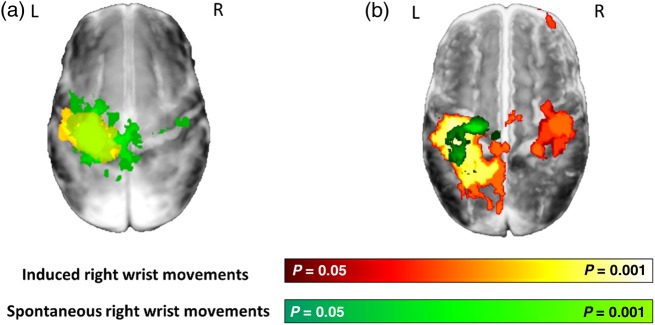
Maturation of functional brain activity associated with spontaneous motor behavior in infants during the preterm period and at term equivalent age. (*a*) Spontaneous movements of the right wrist in preterm infants (median age 33 + 3 weeks PMA, *n* = 10) were associated with a large cluster of activity (green) in the bilateral peri-rolandic regions which is nearly identical to that seen in the same infants following induced movement of the right wrist (red–yellow). (*b*) At term equivalent age (median age 41 weeks PMA, *n* = 8), the functional activity associated with spontaneous movements (green) is more focal and anterior to that seen following somatosensory stimulation (red–yellow).

Spontaneous movements of the right wrist were precisely measured using a fiber-optic goniometer incorporated into the wrist interface, during a separate period of data acquisition in which no movements were directly induced. Although spontaneous motor behavior was recorded in nearly all of infants during the acquisition period (55/62), the majority were also associated with simultaneous head movement. These data were therefore discarded due to the possibility of introducing false correlations through inappropriate interpretation of spikes in the measured BOLD signal caused by head motion (Hajnal et al. 1994; Power et al. 2012; Satterthwaite et al. 2012; Van Dijk et al. 2012). The resultant study group of infants who made isolated right wrist movements therefore consisted of 8 preterm infants (median age 33 + 3 weeks PMA, range: 32 + 2 to 36 + 3 weeks) and 10 infants at term equivalent age (median age 41 + 0 weeks PMA, range: 37 + 5 to 43 + 0 weeks). In all of these infants across both groups, the detected spontaneous right wrist movements were associated with significant increases in the measured BOLD signal in the contralateral (left) peri-rolandic region (Fig. 3). In preterm infants, the associated cluster of functional activity was large and dispersed across the contralateral peri-rolandic cortex and spatially overlaid the cluster identified following passive sensori-motor stimulation (in the same infants), with an additional area of extension into the SMA and ipsilateral peri-rolandic cortex (see Fig. 4). At term equivalent age, the identified cluster of functional activity was similarly located in the contralateral peri-rolandic cortex, but was spatially more localized within a smaller cluster in the anterior portion (comprising the primary motor cortex) in comparison to that identified in the same infants with the passive sensori-motor task.

### Functional Connectivity Analysis

**Figure 5.**
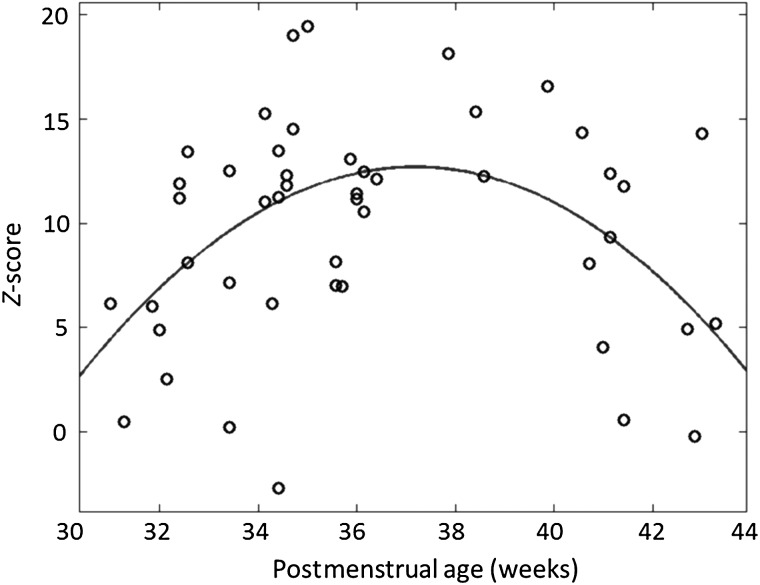
Interhemispheric functional connectivity initially increases rapidly during the preterm period but then appears to decrease at term equivalent age. Partial correlation between the BOLD contrast time-series to somatosensory stimulation in the left and right peri-rolandic regions increases rapidly during the preterm period, and reaches a maximum value at 36 weeks PMA. It then falls at term equivalent age, leading to an inverted U-shaped distribution. This decrease in functional connectivity may result from increasingly specific functional connectivity. (Data represent *z*-transformed Pearson's partial correlation coefficient).

Interhemispheric functional connectivity between the anatomically delineated left and right peri-rolandic cortices during the passive sensori-motor task was seen to markedly increase with maturation during the late preterm period from 31 to 37 weeks PMA (Fig. 5). Connectivity was maximal in the early term equivalent period (37–38 weeks PMA), but there was then a clear trend toward an apparent decrease in connectivity with maturation during the remaining term equivalent period (up to 44 weeks PMA). These trends result in the assessed functional connectivity during a task following an inverted U-shaped distribution across the study period (Fig. 5).

### The Effect of Ex Utero Exposure on Passive Motor Functional Responses

**Figure 6.**
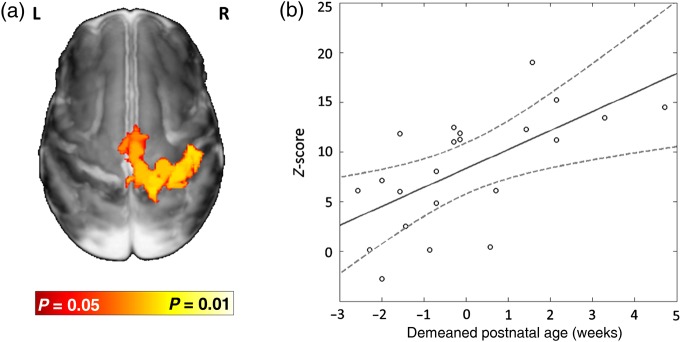
Interhemispheric functional connectivity during the preterm period is influenced by ex utero exposure. (*a*) Up to 35 weeks PMA (*n* = 22), functional activity following right wrist movement in the ipsilateral (right) peri-rolandic cortex is linearly related to the amount of ex utero exposure as measured by postnatal age. Image shows the results of a linear model fitting based on postnatal age at scan (and controlling for PMA) overlaid on an age-specific template. (*b*) In the same infants, functional connectivity between the BOLD signal time-series in the left and right peri-rolandic regions during the task also shows a linear relationship with the demeaned postnatal age.

In preterm infants from 31 to 34 + 6 weeks PMA (*n* = 22), ex utero exposure (as measured by the PNA at scan) was significantly associated with an increasingly bilateral pattern of functional response. This effect was seen first by the increased spatial presence of functional activity within the ipsilateral (right) peri-rolandic cortex and SMA in preterm infants with an older PNA at scan, regardless of their PMA. This relationship was found to be highly significant (*P* < 0.05) and linear (Fig. 6*a*). A significant effect was also seen in the interhemispheric functional connectivity, with the *z*-transformed Pearson's partial correlation coefficient of the relationship between the time-series in the left and right peri-rolandic regions similarly seen to increase in a linear fashion relative to an increase in the PNA in days of preterm infants up to 34 + 6 weeks PMA (Fig. 6*b*). At later PMA (35–44 weeks), the relationship between PNA and the pattern of brain activity was found to no longer reach significance as the overall developmental effect related to PMA appears to predominate, with the majority of infants having a bilateral pattern of response regardless of their PNA.

## Discussion

Using fMRI and an optimized task-based paradigm, we have performed the most comprehensive developmental characterization yet reported of sensori-motor functional activity in the equivalent period to the mid-third trimester of human gestation. We observed systematic maturation of the temporal and spatial properties of the functional responses, interhemispheric functional connectivity, and a significant effect related to postnatal exposure.

### Maturation of Functional Responses and the Sensori-Motor Network

Our results demonstrate that the primary functional response following sensori-motor stimulation is established in the contralateral primary motor and somatosensory cortices from the mid-third trimester, and is transmitted via the classical dorsal column-medial lemniscal and thalamo-cortical pathways (Purves 2012). Over the subsequent 6 weeks, the spatial extent of the primary activity increased, and there was gradual integration of regions associated with mature sensori-motor processing (including the SMA and ipsilateral peri-rolandic regions) (Blatow et al. 2007). Our findings are also in agreement with previous feasibility studies which have similarly found well-localized clusters of contralateral or bilateral BOLD responses using different patterns of somatosensory stimulation (Erberich et al. 2003, 2006; Heep et al. 2009; Arichi et al. 2010). This spatial progression is strikingly reminiscent of RSN maturation, where an initially fragmented representation also progresses to a distinct adult-like topology at term (Doria et al. 2010). This process is likely driven by increases in thalamo-cortical and cortico-cortical connectivity through the transient subplate zone, as its ablation in preterm-equivalent rats eliminates both endogenous and evoked spindle burst activity, and permanently alters barrel cortex histological patterning (Kostovic and Rakic 1990; Kostovic and Jovanov-Milosevic 2006; Tolner et al. 2012). At the end of the third trimester, there is gradual dissolution of the subplate and a rise in the thickness of cortical layer IV (which makes a large contribution to the BOLD functional response due to its high metabolic demand and dense vasculature (Goense et al. 2012)), which coincides with further elaboration of thalamo-cortical axonal connections and intracortical circuitry (Marin-Padilla 1970; Kostovic and Jovanov-Milosevic 2006). These neural factors, in addition to increases in cerebral blood flow and intracerebral vasculature (Greisen 1986; Risser et al. 2009; Roche-Labarbe et al. 2014) may also explain the significant trend toward faster and higher amplitude responses at the end of the third trimester (Arichi et al. 2012).

### Evolution of Functional Activity at Term Equivalent Age

At term, we observed a transition in sensori-motor functional responses, including further integration of regions (including the secondary somatosensory cortex and basal ganglia) and spatial “refinement” with a more significant and localized functional response. Analogous maturation is seen in rats, where initially nonspecific receptive fields become more specific and topographically organized in the equivalent period (Seelke et al. 2012). This may be partly explained by the emergence of cortical inhibitory signaling (Ben-Ari 2002; Ben-Ari et al. 2004). The observed maturational decrease in response amplitude also corresponds to a clear transition in evoked responses demonstrated with electrophysiological techniques; as spatially dispersed and modality-independent high-amplitude delta-brush activity shifts to spatially localized, faster and modality-specific somatosensory evoked potentials (Hrbek et al. 1973; Fabrizi et al. 2011).

The integration of distinct structures into a sensori-motor functional network is thought fundamental to human motor control and behavior in adults, with impairment correlating with poor outcome following stroke (Grefkes et al. 2008; Carter et al. 2012). Although neonatal motor behavior is generally not purposeful (and therefore appears mismatched to the spatial maturity of their brain activity) (Cioni and Prechtl 1990), perinatal injury to structures within this network significantly increases the risk of long-term motor dysfunction; suggesting that the described maturational processes are crucial for establishing life-long sensori-motor function (Juenger et al. 2011).

### Functional Activity Associated with Spontaneous Motor Behavior

By precisely measuring spontaneous wrist movements, we have demonstrated that endogenous motor behavior in young infants is associated with localized functional activity in the sensori-motor cortex. Despite being very settled or sleeping, the studied infants made frequent limb movements; although most were associated with generalized movements of the head and body, necessitating a large amount of data discard. Regardless, we found that the associated activity in preterm infants largely overlapped the induced functional responses in the contralateral peri-rolandic cortex. At term equivalent age, the activity was more spatially specific within a more anteriorly located cluster. In both groups, there was activity in the SMA, which is of particular interest as it plays an essential role in planning movements in the mature brain (Weilke et al. 2001). While we did not monitor movement in other limbs, similar robotic devices can be fitted; thus allowing further characterization of the emergence of functional somatotopy (Allievi et al. 2014).

Infants make frequent spontaneous (but not goal-orientated) movements, with abnormalities in quantity and quality found to predict later neurological dysfunction (Prechtl 1990; Skiold et al. 2013). Limitations inherent to both the fMRI acquisition and the biophysical properties of the BOLD response prevent a definitive interpretation of the activity's role in spontaneous motor behavior, which could be either efferent (generated endogenously and resulting in limb movement) or afferent (processing of information about spinally generated movements) in nature. Animal studies however suggest the latter which is thought to provide crucial ascending somatotopic cortical feedback (O'Donovan 1999; Khazipov et al. 2004; Khazipov and Luhmann 2006). In preterm human infants, high-amplitude bursts of delta-brush neural activity in the contralateral hemisphere are triggered by spontaneous movements, but can also occur endogenously (without a behavioral correlate) or are readily elicited by somatosensory stimulation, further supporting the premise that the movements are initiated subcortically but then provide sensory input essential for cortical development (Milh et al. 2007; Fabrizi et al. 2011; Tolner et al. 2012).

### Developmental Trends and Experience-Dependent Effects on Interhemispheric Functional Connectivity

With increasing PMA, we saw increasing ipsilateral cortical involvement, which coincides with known maturational increases in MR measures of corpus callosal microstructure such as fractional anisotropy (Aeby et al. 2009; Nossin-Manor et al. 2013). Although we saw a counterintuitive decrease in interhemispheric functional connectivity at term, this likely reflects the increased spatial specificity of the response (rather than decreasing connectivity), due to less correlation of the mean BOLD time-series across the entire peri-rolandic regions.

Up to the late preterm period, ipsilateral sensori-motor activity and interhemispheric functional connectivity were significantly related to PNA, suggesting a potential role for experience-dependent processes. This agrees with developmental animal studies which show that early-life sensory experience leads to the strengthening and modification of existing synapses (rather than an increase in synaptogenesis), and can alter long-term somatosensory cortical topography and corpus callosal structure (Bourgeois et al. 1989; Fox 1992; Wang et al. 2007). In this study, we collectively described all sensory stimulation during the preterm period as “ex-utero experience” and, therefore, our findings are only suggestive of an effect as we cannot be certain to what degree other uncontrolled prematurity-related clinical factors (such as sepsis or particular medications) contributed. However, the possible effect may have implications for the management of preterm infants in the NICU as they are frequently exposed to adverse psycho-physiological stimuli (such as stress and pain) as part of their routine clinical care (Brummelte et al. 2012; Ranger et al. 2013). Conversely, the mid–late-third trimester may also represent a potential window of positively influencing brain development to improve preterm outcome and treat perinatally acquired brain injury (Als et al. 2004; Guzzetta et al. 2011). It is therefore crucial that this possible effect is carefully tested in further prospective and longitudinal studies; which can quantitatively assess both the effects of specific experiences (both directly induced and through clinical care) and importantly whether the changes are sustained (and correlate with clinical outcome) in later life.

### Limitations and Further Work

Our study group consisted predominately of preterm infants, and thus our findings may only be representative of functional development specific to this group. While we limited our study population to “healthy” preterm infants and all of the infants at term equivalent age had a mild or moderately abnormal appearance to the white matter (and therefore not highly predictive of later cerebral palsy), we cannot exclude the presence of microstructural lesions in our study population as prematurity is commonly associated with subtle white matter injury that may not be apparent on qualitative assessment of structural MRI images alone. Future work could therefore also greatly benefit from correlating maturational fMRI data with diffusion MRI quantitative measures of microstructural integrity, particularly within vulnerable structures such as the corpus callosum (Zhang et al. 2012). To avoid the confounding effects of head motion, we discarded data and administered a light sedative to most term infants. While in this study in a small subset of subjects we did not find that sedation affected functional activity; there is a need for a larger and systematic study of the specific effects of sedation on BOLD responses in infants as there is also recent work suggesting that it may decrease response amplitude (Williams et al. 2015). Future studies will also benefit from removing this potential bias through applying motion-tolerant data acquisition and analysis techniques (Maclaren et al. 2013; Ferrazzi et al. 2014).

## Conclusions

The sensori-motor system rapidly but systematically matures in the human brain during the preterm period (equivalent to the mid- to late-third trimester), and may be significantly influenced by experience. Fundamental to this is the integration of distinct regions which comprise the sensori-motor functional network, with a spatially specific and mature distribution evident at term equivalent age. Our findings highlight the importance of this period for the establishment of life-long sensori-motor function, and provide a foundation for studies of the specific effects of pathology and therapies.

## Supplementary Material

Supplementary material can be found at: http://www.cercor.oxfordjournals.org/.

## Authors’ Contributions

A.G.A. and T.A. made equal contributions to the work, and were involved in all aspects including: study design, data collection, data analysis and interpretation, and manuscript preparation. N.T. and S.J.C. were involved in data collection and interpretation, and reviewed the manuscript. J.K. and S.A. were involved in data analysis and reviewed the manuscript. A.D.E. and E.B. were involved in the study design and preparation, data interpretation, and manuscript preparation.

## Funding

The authors acknowledge support from the Department of Health via the National Institute for Health Research (NIHR) comprehensive Biomedical Research Centre award to Guy's & St Thomas’ NHS Foundation Trust in partnership with King's College London and King's College Hospital NHS Foundation Trust. A.G.A. and T.A. were supported by funding from the Engineering and Physical Sciences Council UK (EPSRC) and National Institute of Health Research UK (NIHR), respectively. A.G.A. and E.B. were supported in part by the EU FP7 grants PEOPLE-2012-ITN 317488 CONTEST, ICT-2013 601003 BALANCE, ICT-2013 SYMBITRON 611626, and the H2020 grant COGIMON ICT-23-2014 644727. Funding to pay the Open Access publication charges for this article was provided by the Commission of the European Communities.

## Supplementary Material

Supplementary Data
