# Supplementary material for: Maturation of Sensori-Motor Functional Responses in the Preterm Brain
Source: Cereb Cortex. 2015 Oct 21;26(1):402–13. doi: 10.1093/cercor/bhv203 (PMC4677983; doi:10.1093/cercor/bhv203)
Supplement: Supplementary Data [file supp_26_1_402__index.html]

Maturation of Sensori-Motor Functional Responses in the Preterm Brain — Maturation of Sensori-Motor Functional Responses in the Preterm Brain — Supplementary Data 

# Maturation of Sensori-Motor Functional Responses in the Preterm Brain

## Supplementary Data

Supplementary Data

- Supplementary Data - Docx file
- Supplementary Video 1 - mp4 file
