# Supplementary material for: Maturation of Sensori-Motor Functional Responses in the Preterm Brain
Source: Cereb Cortex. 2015 Oct 21;26(1):402–13. doi: 10.1093/cercor/bhv203 (PMC4677983; doi:10.1093/cercor/bhv203)
Supplement: Supplementary Data [file supp_bhv203_bhv203supp.docx]

**Movie 1:** Fully automated and fMRI compatible wrist robot used for motor stimulation in the study.
